# CD4 Cell Count and the Risk of AIDS or Death in HIV-Infected Adults on Combination Antiretroviral Therapy with a Suppressed Viral Load: A Longitudinal Cohort Study from COHERE

**DOI:** 10.1371/journal.pmed.1001194

**Published:** 2012-03-20

**Authors:** The Opportunistic Infections Project Team of the Collaboration of Observational HIV Epidemiological Research in Europe (COHERE) in EuroCoord

**Affiliations:** Duke University Medical Center, United States of America

## Abstract

**Background:**

Most adults infected with HIV achieve viral suppression within a year of starting combination antiretroviral therapy (cART). It is important to understand the risk of AIDS events or death for patients with a suppressed viral load.

**Methods and Findings:**

Using data from the Collaboration of Observational HIV Epidemiological Research Europe (2010 merger), we assessed the risk of a new AIDS-defining event or death in successfully treated patients. We accumulated episodes of viral suppression for each patient while on cART, each episode beginning with the second of two consecutive plasma viral load measurements <50 copies/µl and ending with either a measurement >500 copies/µl, the first of two consecutive measurements between 50–500 copies/µl, cART interruption or administrative censoring. We used stratified multivariate Cox models to estimate the association between time updated CD4 cell count and a new AIDS event or death or death alone. 75,336 patients contributed 104,265 suppression episodes and were suppressed while on cART for a median 2.7 years. The mortality rate was 4.8 per 1,000 years of viral suppression. A higher CD4 cell count was always associated with a reduced risk of a new AIDS event or death; with a hazard ratio per 100 cells/µl (95% CI) of: 0.35 (0.30–0.40) for counts <200 cells/µl, 0.81 (0.71–0.92) for counts 200 to <350 cells/µl, 0.74 (0.66–0.83) for counts 350 to <500 cells/µl, and 0.96 (0.92–0.99) for counts ≥500 cells/µl. A higher CD4 cell count became even more beneficial over time for patients with CD4 cell counts <200 cells/µl.

**Conclusions:**

Despite the low mortality rate, the risk of a new AIDS event or death follows a CD4 cell count gradient in patients with viral suppression. A higher CD4 cell count was associated with the greatest benefit for patients with a CD4 cell count <200 cells/µl but still some slight benefit for those with a CD4 cell count ≥500 cells/µl.

*Please see later in the article for the Editors' Summary*

## Abstract 2

Using data from the Collaboration of Observational HIV Epidemiological Research Europe, Jim Young and colleagues show that in successfully treated patients the risk of a new AIDS event or death follows a CD4 cell count gradient in patients with viral suppression.

## Editors' Summary

**Background:**

Currently, about 34 million people are infected with HIV and every year nearly 3 million people are newly infected with this virus, which causes AIDS. Most people do not become ill immediately after infection with HIV although some develop a short, flu-like illness (a “seroconversion” illness). The next stage of HIV infection, which may last up to 10 years, also has no major symptoms but, during this stage, HIV slowly destroys immune system cells (including CD4 cells, a type of lymphocyte). Eventually, the immune system can no longer fight off infections by other disease-causing organisms and HIV-positive people then develop one or more AIDS-defining condition(s), including severe but unusual infections, Kaposi sarcoma (a skin cancer), and non-Hodgkin lymphoma (a cancer of the lymph nodes). Many of these AIDS-defining conditions are life-threatening and, in the past, HIV-positive people died on average within 10 years of infection. Nowadays, although there is still no cure for HIV infection, combination antiretroviral therapy (cART; a cocktail of powerful antiretroviral drugs) has turned HIV/AIDS into a chronic, treatable condition, at least in developed countries.

**Why Was This Study Done?:**

Most HIV-positive adults achieve viral suppression within a year of starting cART. That is, the number of copies of the virus in their blood drops to below 50 copies/ml. But what is the likely clinical outcome for patients who achieve viral suppression and what is their risk of developing a new AIDS-defining condition or of dying? For people starting cART for the first time, the number of CD4 cells in the blood when cART is initiated provides a strong indication of an individual's likely clinical outcome. Specifically, people who start cART when they have a high CD4 cell count tend to do better than people who start treatment when they have a low CD4 cell count. In this study, the researchers use data collected by the Collaboration of Observational HIV Epidemiological Research in Europe (COHERE) to estimate the association between CD4 cell count and progression to a new AIDS-defining event or death among patients who have achieved viral suppression while on cART.

**What Did the Researchers Do and Find?:**

The researchers identified more than 75,000 patients in the COHERE database who, between them, had had more than 104,000 episodes (periods) of viral suppression while on cART and who had had their CD4 cell count determined shortly before or during their viral suppression episodes. The researchers then used stratified multivariate Cox models (a type of statistical analysis method) to estimate the association between CD4 cell counts and the occurrence of a new AIDS-defining event or death. Among the patients included in the study, the mortality (death) rate was 4.8 per 1,000 years of viral suppression. The highest rates of new AIDS-defining events or death were seen in those patients with less than 50 CD4 cells/µl blood and a higher CD4 cell count was associated with a reduced risk of a new AIDS-defining event or death. Finally, among those patients with a CD4 cell count below 200 cells/µl, the risk of progression decreased over time for those patients with higher CD4 cell counts.

**What Do These Findings Mean?:**

These findings suggest that, although new AIDS-defining events and death are uncommon among patients whose viral load is suppressed by cART, the risk of a new AIDS-defining event or death follows a CD4 cell count gradient with the patients with the highest CD4 cell counts having the lowest risk of a new AIDS-defining event or death. The findings also suggest that higher CD4 cell counts provide the greatest benefit for patients with a CD4 cell count below 200 cells/µl blood. These findings have two main clinical implications. First, they add to the evidence that suggests that, to facilitate immune system recovery, cART should be started when a patient's CD4 cell count is between 350 and 500 cells/µl blood, the current recommended range for cART initiation. Unfortunately, most patients in resource-limited settings only start cART when their CD4 cell count is below 200 cells/µl. Second, these findings suggest that patients with sustained viral suppression but low CD4 cell counts should be monitored regularly to ensure that any life-threatening AIDS-defining events are dealt with quickly and effectively.

**Additional Information:**

Please access these Web sites via the online version of this summary at http://dx.doi.org/10.1371/journal.pmed.1001194.

- Information is available from the US National Institute of Allergy and infectious diseases on HIV infection and AIDS
- NAM/aidsmap provides basic information about HIV/AIDS, and summaries of recent research findings on HIV care and treatment
- Information is available from Avert, an international AIDS charity on many aspects of HIV/AIDS, including detailed information on HIV treatment and care (in English and Spanish)
- The World Health Organization's 2010 antiretroviral therapy guidelines provide recommendations on when to initiate cART
- Information about COHERE is available
- Patient stories about living with HIV/AIDS are available through Avert and through the charity website Healthtalkonline

## Introduction

More than 90% of those infected with HIV now achieve viral suppression within a year of starting a combination antiretroviral therapy (cART) [1],[2]. Patients with a suppressed viral load now represent the majority of cART recipients. Previous cohort studies have shown that the CD4 cell count when starting cART is the most important prognostic factor for clinical outcome, but these studies have focused on cART-naïve patients and have ignored treatment changes and periods of detectable viral load [3],[4].

This study considers the prognostic value of a CD4 cell count, not when starting cART, but while a patient is being successfully treated, that is, while a patient is on cART with a suppressed viral load. For many patients, viral suppression is not continuous but episodic with periods of viremia as a result of treatment interruption or treatment failure. We selected patients from the Collaboration of Observational HIV Epidemiological Research in Europe (COHERE) database, accumulated episodes of viral suppression for each patient while on cART, and used these episodes to estimate the association between a time updated CD4 cell count and progression to a new AIDS-defining event or death, or death alone.

## Methods

### The COHERE Collaboration

COHERE is a collaboration of European HIV cohorts (http://www.cohere.org/). The 22 cohorts participating in this project provided data in a standardised format to one of two regional co-ordinating centres, where basic error checks were carried out and duplicate records removed for patients followed in more than one cohort. Data collected included information on patient characteristics, antiretroviral therapy, CD4 cell count, HIV RNA viral load, AIDS events, and causes of death. This analysis was based on data merged in 2010 when, for the first time, additional data were collected on co-infection with and treatment for hepatitis B or C, and on the prophylaxis and treatment of opportunistic infections. Patients included in the 2010 merger had to have this additional information and follow-up after 1 January 1997.

### Patient Inclusion

Patients were eligible for our analyses if they achieved one or more episodes of viral suppression while on cART. Optimal viral suppression is defined as a viral load below the level of detection or below 20–75 copies/µl depending on the assay used; however, isolated transient detectable viral loads below 400 copies/µl are not uncommon in successfully treated patients and are not thought to represent an increased risk of virologic failure [5]. We defined the start of a suppression episode as the second of two consecutive viral load measurements below 50 copies/µl (or below the limit of detection) while on cART. We defined the end of a suppression episode as a viral load measurement below 50 copies/µl (or undetectable) then followed by either (1) a measurement greater than 500 copies/µl, (2) the first of two consecutive measurements between 50 and 500 copies/µl, (3) an interruption in cART, or (4) no further viral load measurements. Note that our definition allows for isolated viral load measurements of between 50 and 500 copies/µl within a suppression episode. We defined cART as any three antiretroviral drugs from any drug class, except that three nucleoside (or nucleotide) reverse-transcriptase inhibitors (NRTIs) was only considered cART if taken after another cART regimen.

Patients with at least one suppression episode were then included in our time to event analyses if pre-specified covariates were also available. Patients had to have a CD4 cell count measured within 6 mo prior to the start of an episode or within an episode, and CD4 cell counts were updated over time in our analyses so that each episode was represented by a set of intervals, one interval per CD4 cell count, using the counting process method of representing time to event data. Patients with more than one suppression episode contributed more than one set of intervals to our analyses, but were not at risk between episodes (see [6]). We deleted any interval where the CD4 cell count was measured before the patient was 16 y old. Other covariates were age (in the year 2000), gender, intravenous drug use as the likely mode of HIV transmission, viral load, co-infection with hepatitis B or C, cART category, and the number of prior cART regimens, with these last three covariates updated for each interval. For a first suppression episode, we used a last viral load prior to starting cART as the viral load covariate; for a subsequent episode, we used the highest viral load between the previous and current episode as the covariate.

### Statistical Methods

Our primary outcome was time to a first new AIDS event or death while suppressed and on cART, with an AIDS event defined as one of the conditions listed in Appendix B of the 1993 US Centers for Disease Control (CDC) AIDS surveillance case definition [7]. We used Cox proportional hazards models to estimate the association between an AIDS event or death and CD4 cell count, with CD4 cell count represented by a linear spline with three knots at 200, 350, and 500 cells/µl [8]. These knots correspond to thresholds in treatment guidelines below; below these three thresholds, antiretroviral treatment is essential, recommended, or should be considered, respectively [5]. A hazard ratio (HR) <1.0 for any of the four components of this spline implies that a higher CD4 cell count (per 100 cells/µl) is associated with a lower risk of progression and is therefore a measure of the benefit that a patient can expect if their CD4 cell count increases above any current level within the range covered by that spline component. Our models included the baseline and time updated covariates described above. We stratified our models by cohort, so that each cohort had its own non-parametric baseline hazard function, but we assumed the effect of each covariate was the same in each cohort [9]. To assess whether the hazards associated with CD4 cell count were constant over time (i.e., proportional hazards), we fitted a model with interaction terms between log suppression time and CD4 cell count, with these interactions centred around the geometric mean suppression time [10]–[12].

We carried out six planned sensitivity analyses to check that our estimates were stable. Assays have become more sensitive over time, so we re-fitted our model with (1) a suppression episode re-defined as a viral load below 400 copies/µl—to simulate constant use over time of a less sensitive assay; and (2) with the analysis restricted to suppression episodes starting after 1 January 2001—to largely omit episodes found using less sensitive assays [13]. We varied the period of time after a last viral load measurement within which new AIDS events or death were accepted as outcomes if suppression was ongoing. We considered such events as outcomes if they occurred within 180 d of a last viral load where the patient was still suppressed at this last measurement, but in sensitivity analyses we re-fitted our model assuming (3) shorter and (4) longer periods (90 and 270 d, respectively). We dropped covariates from our model to retain episodes lost from our analyses because of missing covariates. We re-fitted our model (5) without viral load as a covariate because for many patients, we did not have a viral load measured prior to starting cART; and (6) without co-infection with hepatitis as a covariate, because then we could include additional patients in our analysis from the 2008 merger of the COHERE database [14]. Finally in a single unplanned sensitivity analysis, we assessed whether the risk of progression differed between first and subsequent episodes of viral suppression. We added an additional covariate to the analysis of the primary outcome, either taking value zero for a first suppression episode and one otherwise, or taking value zero for a first suppression episode and the number of years between successive episodes otherwise.

Our secondary outcome was time to death while both suppressed and on cART. We classified a death as “related to HIV” if death was attributed at least in part to an “AIDS defining event” or an “invasive bacterial infection.” If these two causes were not mentioned but other causes of death were given, we classified a death as “unrelated to HIV.” If no causes of death were given, we classified a death as of “unknown cause.” We then fitted a Cox model with different cause-specific hazards for CD4 cell count [15], again with CD4 cell count represented by a linear spline and with the same covariates as before.

Analyses were carried out with the PHREG procedure in SAS version 9.2; survival curves were plotted with the Survival package version 2.36-2 in R version 2.12.1. We report model estimates as HRs, each with a 95% CI.

## Results

### Patient Characteristics

Of the 176,585 patients in the 2010 merger of COHERE, 75,336 patients provided 104,265 suppression episodes while on cART (); 71% of these patients had just a single episode. The median length of a suppression episode was 1.7 y (interquartile range [IQR] 0.7–3.5); the median total time suppressed while on cART was 2.7 y (IQR 1.2–5.1) per patient; the estimated average gain in CD4 cell count while suppressed was 53 cells/µl per year. The main analyses of primary and secondary outcomes were based on 66,147 patients with a viral load measured prior to starting cART. Patients contributing to our main analyses tended to be slightly older, were less likely to be either female or infected through drug use, and were more likely to be recorded as of European origin than other patients in this merger of COHERE (Table 1). Few patients (1%) started their first suppression episode with a CD4 cell count below 50 cells/µl and many (34%) started with a CD4 cell count above 500 cells/µl.

**Table 1:** COHERE patients with continuous or episodic viral suppression while on cART.

Patient Characteristics	Included (*n* = 66,147)	Excluded (*n* = 110,438)
Age at 2,000, y, median (IQR)	37 (32–44)	35 (28–41)
Percent female	27	29
Percent ever diagnosed with AIDS	26	25
Percent transmission by drug use	14	17
Percent recorded as of European nationality^a^	43	34
Year first suppression episode began, median (IQR)	2003 (2000–2006)	
CD4 cell count, cells/µl, median (IQR)^b^	396 (256–565)	
HIV RNA viral load, log 10 copies/ml, median (IQR)^c^	4.6 (3.5–5.2)	
Percent hepatitis B or C^b^	9	
Percent cART category^b^		
NNRTI	34	
PI boosted with ritonavir	30	
PI without ritonavir	25	
Other^d^	11	
Percent CD4 cell category, cells/µl^b^		
<50	1	
50 to <200	15	
200 to <350	26	
350 to <500	25	
≥500	34	

Note that the number of patients included in the main analyses of primary and secondary outcomes (*n* = 66,147) is lower than the number of patients in Tables 2–4 with at least one suppression episode (*n* = 75,336) because viral load prior to starting cART was not known for some patients.

a Nationality was not recorded for 40% of patients included in these analyses and for 50% of patients excluded from these analyses.

b At the start of a first suppression episode. Note that 103 patients that did not contribute a first episode to the main analyses because either no CD4 cell counts were available for this episode or this episode occurred while the patient was still under the age of 16.

c Last viral load before starting combination therapy. Note that a further 4,211 patients did not contribute a first episode to the main analyses because their viral load before starting combination therapy was not known (although these patients contributed a first episode to the sensitivity analysis without this covariate).

d Other: at least one protease inhibitor (PI) and one non-nucleoside reverse-transcriptase inhibitor (NNRTI), 5%; three NRTIs, 3%; at least two PIs (other than ritonavir) but no NNRTI, 2%; any therapy including integrase or fusion inhibitors, 1%.

### Event Rates

The rate of progression to a first new AIDS event or death was 8.9 per 1,000 y of suppression; the mortality rate was 4.8 per 1,000 y of suppression. Both rates showed a gradient that depends on CD4 cell count with the highest rates in those with <50 CD4 cells/µl at the time of the event (Table 2). Even mortality from causes thought unrelated to HIV (Table 2) and the rate of HIV related neoplasms (Table 3) increased with decreasing CD4 cell count. The rate of progression to a first new AIDS event or death decreased over time in all CD4 strata (Table 4), except where patients had a low CD4 cell count (0 to <200 CD4 cells/µl).

**Table 2:** Event rates in CD4 strata among the 75,336 patients with at least one suppression episode while on cART: event rates per 1,000 y of suppressed viral load (number of events) by outcome.

Most Recent CD4 Cell Count (Cells/µl)	First New AIDS Event or Death from Any Cause		Death from Any Cause		Death from Causes Unrelated to HIV	
<50	94.9	(54)	64.8	(38)	25.6	(15)
50 to <200	30.5	(489)	20.0	(325)	14.1	(230)
200 to <350	12.0	(548)	6.9	(318)	5.2	(240)
350 to <500	7.9	(487)	3.8	(240)	2.9	(184)
≥500	5.2	(679)	2.4	(315)	1.9	(253)

**Table 3 pmed-1001194-t003:** Event rates in CD4 strata among the 75,336 patients with at least one suppression episode while on cART: event rates per 1,000 y of suppressed viral load (number of events) for a first new AIDS event, with each event then classified as either an opportunistic infection or a HIV related neoplasm.

Most Recent CD4 Cell Count (Cells/µl)	First New AIDS Event		Opportunistic Infection		HIV Related Neoplasm	
<50	29.9	(17)	21.1	(12)	8.8	(5)
50 to <200	10.8	(173)	7.5	(121)	3.2	(51)
200 to <350	5.2	(239)	3.4	(155)	1.8	(82)
350 to <500	4.0	(249)	2.5	(153)	1.6	(96)
≥500	2.9	(376)	2.0	(256)	0.9	(119)

Four first new AIDS events could not be classified as either an opportunistic infection or an HIV-related neoplasm.

**Table 4:** Event rates in CD4 strata among the 75,336 patients with at least one suppression episode while on cART: event rates per 1,000 y of suppressed viral load (number of events) over time for the primary outcome (a first new AIDS event or death).

Most Recent CD4 Cell Count (Cells/µl)	<1 y		1 to <2 y		≥2 y	
<50	108.5	(36)	73.2	(7)	77.6	(11)
50 to <200	35.4	(251)	22.7	(89)	29.7	(149)
200 to <350	18.2	(247)	9.8	(108)	9.2	(193)
350 to <500	11.5	(160)	7.6	(105)	6.5	(222)
≥500	8.7	(173)	5.3	(128)	4.4	(378)

**Figure 1:**
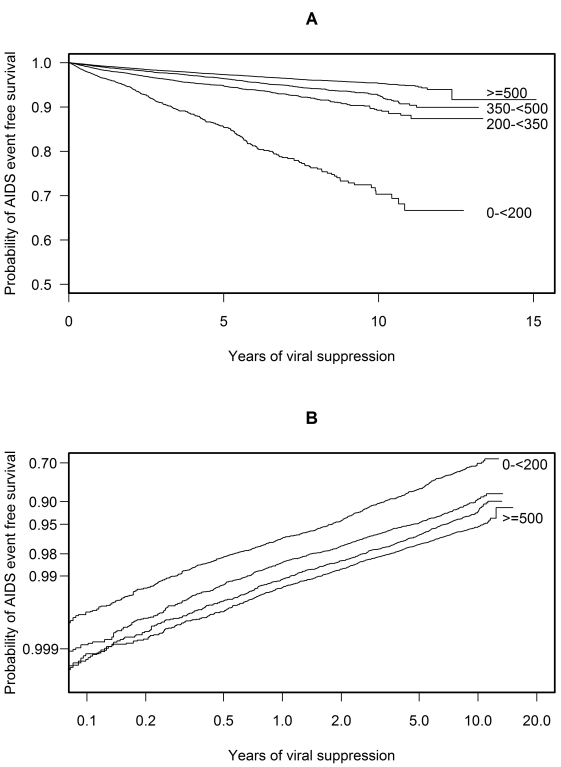
Probability plots of AIDS event-free survival over time. These plots apply to hypothetical patients whose CD4 cell count remains within the same CD4 stratum while on cART with a suppressed viral load. Plot (A) shows a Kaplan Meier plot of the probability of AIDS event-free survival over time. Plot (B) shows a plot of log(−log [probability of AIDS event-free survival]) against log(time). The roughly parallel lines of plot (B) suggest that a proportional hazards model is appropriate for these data. Both plots use a method appropriate for a time-dependent CD4 cell count (see [16]).

A time updated Kaplan Meier plot illustrates the relatively low probability of AIDS event-free survival—roughly 70% after 10 y of suppression—should a patient's CD4 cell count remain below 200 cells/µl while suppressed (Figure 1A) [16]. In contrast, the probability of AIDS event-free survival was roughly 95% after 10 y of suppression for patients maintaining a CD4 cell count of 500 cells/µl or more while suppressed. It is important to note that CD4 cell count was time dependent and updated when calculating these probabilities. Therefore this plot shows probabilities for hypothetical patients whose CD4 count remains within the same CD4 stratum while suppressed [16]. The roughly parallel lines in the plot of AIDS event-free survival (log log scale) against time (log scale) suggest that a proportional hazards model was appropriate for these data (Figure 1B) [11],[12].

### Time to AIDS or Death

A Cox proportional hazards model for time to a first new AIDS event or death also showed a gradient that depends on CD4 cell count (Table 5). A higher CD4 cell count was associated with a much greater decrease in the risk of progression when a patient had a CD4 cell count below 200 cells/µl (HR 0.35, 0.30–0.40, per 100 cells/µl) than when a patient had a CD4 cell count above 500 cells/µl. However, even at a CD4 cell count above 500 cells/µl, a higher CD4 cell count was associated with a slightly reduced risk of progression (HR 0.96, 0.92–0.99, per 100 cells/µl). A higher CD4 cell count had intermediate benefit for CD4 cell counts in the range from 200 to 350 and from 350 to 500 cells/µl.

**Table 5:** HR estimates and their 95% CIs from multivariate Cox proportional hazard models for both the primary and secondary outcome in 66,147 patients on cART with a suppressed viral load.

Model Parameter	Primary Outcome: Time to a First New AIDS Event or Death (1,838 Events)		Secondary Outcome: Time to Death from Any Cause (1,000 Events)	
	HR	95% CI	HR	95% CI
Age (per 10 y)	1.42	1.36–1.49	1.80	1.70–1.91
Female	0.99	0.88–1.11	0.77	0.65–0.90
Transmission by drug use	1.95	1.73–2.20	2.86	2.45–3.33
Hepatitis B or C^a^	1.26	1.05–1.51	1.44	1.11–1.88
Number of prior cART regimens^a^	0.99	0.97–1.02	1.02	0.99–1.05
HIV RNA (per log 10 copies)^b^	1.02	0.98–1.06	1.00	0.95–1.05
cART (with NNRTI as the reference category)^a^				
Three NRTIs	1.14	0.93–1.39	1.36	1.06–1.76
PI without ritonavir	1.08	0.93–1.25	1.19	0.98–1.46
PI boosted with ritonavir	1.17	1.04–1.32	1.13	0.95–1.33
Other^c^	1.25	1.05–1.48	1.37	1.10–1.71
CD4 cell count (per 100 cells/µl) as a linear spline^d^				
0 to <200	0.35	0.30–0.40	0.32	0.27–0.39
200 to <350	0.81	0.71–0.92	0.75	0.63–0.89
350 to <500	0.74	0.66–0.83	0.68	0.58–0.80
≥500	0.96	0.92–0.99	0.98	0.93–1.03

a Time-dependent covariate.

b Last viral load prior to starting cART or highest viral load recorded between episodes.

c Other: at least one protease inhibitor (PI) and one non-nucleoside reverse-transcriptase inhibitor (NNRTI), at least two PIs (other than ritonavir) but no NNRTI; any therapy including integrase or fusion inhibitors.

d Time-dependent covariate. A HR<1.0 for any of the four components of this spline implies that a higher CD4 cell count (per 100 cells/µl) is associated with a lower risk of progression and is therefore a measure of the benefit that a patient can expect if their CD4 cell count increases above any current level within the range covered by that spline component.

The same model suggests that progression was more likely for older patients (HR 1.42, 1.36–1.49, per 10 y), for those infected by drug use (HR 1.95, 1.73–2.2), and for those with hepatitis B or C (HR 1.26, 1.05–1.51). Progression was also more likely for patients on cART regimens typically used after virologic failure (boosted protease inhibitor or other cART) compared to non-NRTI-based cART (the reference category).

Plots of weighted Schoenfeld residuals (not shown) suggest a proportional hazards assumption was reasonable for these data [11]. However we also fitted a reduced model, with CD4 cell count represented by a linear spine with a just single knot at 200 cells/µl and with interaction terms between each of the two components of this spline and log suppression time. For patients with a CD4 cell count below 200 cells/µl (HR 0.21, 0.19–0.24, per 100 cells/µl), the interaction (HR 0.51, 0.48–0.54) implied that with a higher CD4 cell count, the risk of progression was not constant but decreased over time. For patients with a CD4 cell count above 200 cells/µl (HR 0.92, 0.90–0.94, per 100 cells/µl), the interaction (HR 1.02, 1.00–1.05) implied that the risk of progression was constant over time. The increasing benefit over time of a higher CD4 cell count for patients with low CD4 cell counts is consistent with the increased event rate after 2 y in Table 4 for patients with low CD4 cell counts and the slight increase in slope after 2 y in Figure 1B for patients with low CD4 cell counts.

HRs for the spline representing CD4 cell count were similar in all six planned sensitivity analyses (Text S1). In the unplanned sensitivity analysis, there was no evidence that the risk of progression differed between first and subsequent episodes of viral suppression (Text S1). The estimated average loss in CD4 cell count between the end of one suppression episode and the beginning of the next was 23 cells/µl per year.

### Time to Death

A Cox proportional hazards model for time to death from any cause showed a similar gradient with respect to CD4 cell count (Table 5). There was, however, no real benefit in a higher CD4 cell count for patients with a CD4 cell count above 500 cells/µl (HR 0.98, 0.93–1.03, per 100 cells/µl). And, unlike the primary outcome, women had a lower risk of death (HR 0.77, 0.65–0.90) and cART with three NRTIs was associated with a higher risk of death (HR 1.36, 1.06–1.76).

In a competing risks analysis, we fitted a reduced model with CD4 cell count represented by a linear spine with a just single knot at 200 cells/µl. For patients with a CD4 cell count below 200 cells/µl, a higher CD4 cell count had the most benefit for deaths attributed at least in part to HIV and for deaths of unknown cause (HR 0.20, 0.14–0.30, and 0.22, 0.15–0.32, per 100 cells/µl, respectively), but still had appreciable benefit for deaths thought unrelated to HIV (HR 0.32, 0.26–0.38, per 100 cells/µl). For patients with a CD4 cell count above 200 cells/µl, a higher CD4 cell count had the most benefit for deaths attributed at least in part to HIV (HR 0.58, 0.49–0.70, per 100 cells/µl), but still had some benefit for deaths of unknown cause and for deaths thought unrelated to HIV (HR 0.86, 0.79–0.94, and 0.88, 0.85–0.91, per 100 cells/µl, respectively).

## Discussion

This study shows that a higher CD4 cell count is associated with a reduced risk of clinical progression in patients on cART with a suppressed viral load. For patients with a low CD4 cell count, a higher CD4 cell count becomes even more beneficial over time. The benefits associated with a higher CD4 cell count are similar for patients with a CD4 cell count either between 200 and 350 cells/µl or between 350 and 500 cells/µl. Even patients with a CD4 cell count above 500 cells/µl will benefit to a slight extent from a higher CD4 cell count, although there is little if any association between this and the risk of death. Absolute risk reductions in this highest CD4 cell category, however, will be small at best and of little clinical relevance for most patients.

The benefits seen here appear to apply irrespective of whether viral suppression is continuous or episodic. Additional results from the unplanned sensitivity analysis suggest that, having adjusted for other covariates (including a time updated CD4 cell count), patients with episodic suppression were no more likely to progress than patients with continuous suppression. This does not imply that a period of viremia is without negative consequences. Rather these results are consistent with immunological and epidemiological evidence that the negative consequences of viremia are damage to the immune system and a subsequent decline in CD4 cell count [17]–[20]. For those patients with more than one episode of viral suppression, the estimated loss in CD4 cell count between the end of one suppression episode and the beginning of the next was 23 cells/µl per year.

Our estimates of the benefit associated with a higher CD4 cell count have relatively narrow CIs, are robust across sensitivity analyses, and show logical differences between different outcomes and different causes of death. Although many patients were excluded from this merger of COHERE or from the main analysis because of missing covariate information, sensitivity analyses without these covariates and with these patients included suggest that these exclusions have not had a material effect on estimates. We used time updated CD4 cell count to model the risk of progression because in clinical practice decisions are based on the most recent data [10],[21]. We would underestimate the benefit of a higher CD4 cell count were we to base an analysis on the CD4 cell count at the beginning of a suppression episode because of the decay over time in the predictive value of a first observation [10],[19],[22]. Nevertheless we may still underestimate the benefit associated with a higher CD4 cell count to some extent, possibly because of infrequent updating in some patients but more likely because of the considerable measurement error in CD4 cell counts [21],[23],[24]. We did not adjust for primary prophylaxis as this is on a causal pathway between a low CD4 cell count and outcome (see [25]). The use of prophylactic drugs will result in an underestimate of the benefit associated with a higher CD4 cell count for patients with a low CD4 cell count relative to the benefit one would expect in the absence of any prophylaxis.

Previous studies have shown an increased risk of AIDS or death with lower time updated CD4 cell count in untreated patients and in treatment experienced patients [19],[26], and with lower CD4 cell count at the start of treatment or after 6 mo of treatment in treatment-naive patients [3],[4],[22]. In all these studies, CD4 cell count was the strongest prognostic factor for disease progression; viral load was at best only weakly predictive of progression in models with time updated CD4 cell counts [19],[21]. Here we show an increased risk of AIDS or death with lower time updated CD4 cell count in successfully treated patients. The mortality rate in this study was 4.8 per 1,000 y of suppression; lower than the rate of 12 or 14 per 1,000 y in treatment-naive patients starting cART [3],[27]. The event rates in Tables 2–4 show that CD4 cell count gradients are seen in unadjusted rates; otherwise these rates are of limited value to clinicians because of differences between cohorts in rates of AIDS and death, with differences probably due to different methods of diagnosing disease and ascertaining death [28]. However the association between CD4 cell count and AIDS or death appears much more stable across cohorts [28], consistent with our analytic approach where each cohort had a separate baseline hazard but covariate effects were assumed to be the same in each cohort.

The results of this study provide further indirect evidence for starting cART when a patient's CD4 cell count is between 350 and 500 cells/µl [29],[30]. In this study the benefits associated with a higher CD4 cell count were similar over a range of CD4 cell counts from 200 to 500 cells/µl. Above a count of 500 cells/µl, a higher CD4 cell count was associated with a slightly reduced risk of an AIDS event but had little association with the risk of death; hence even earlier treatment with a CD4 cell count above 500 cells/µl might be appropriate for patients with characteristics associated with slower immune recovery—older patients, those with a drug addiction, or co-infected with viral hepatitis [31]–[33]; such patients had a greater risk of progression in our study. A higher CD4 cell count was also associated with a reduced risk of death from causes thought unrelated to HIV. This finding suggests that the distinction between causes of death related and unrelated to HIV is rather arbitrary in successfully treated patients, and that there is a need for more sophisticated recording and review of causes of death to avoid underestimating the burden of HIV infection [28],[34],[35].

In several studies, a CD4 cell count of around 200 cells/µl has been seen as an important threshold [3],[21],[22]. The strength of time updated CD4 cell count as a prognostic factor for survival has led to a suggestion that “there is a threshold beyond which immune reconstitution may be compromised” [22]. Others argue that patients starting treatment with low counts do not seem to remain disadvantaged if the CD4 cell count at the start of treatment is not predictive of survival once adjusted for a value at 6 mo [4]. We see our results—with a higher CD4 cell count becoming even more important over time for patients with low CD4 cell counts—as more consistent with the idea of lasting damage below some threshold from which recovery is difficult [18],[36]. Many patients starting therapy with a CD4 cell count below 200 cells/µl never achieve a normal CD4 cell count even after 10 y of otherwise effective antiretroviral therapy [37], although this failure to recover could be due to factors other than a low CD4 cell count per se. Various treatment intensification strategies have failed to show any benefit in patients with low CD4 cell counts [38],[39]. Despite improvements, the majority of patients in resource-limited settings still start therapy with a CD4 cell count below 200 cells/µl [40], so that along with improved access to treatment, earlier diagnosis and earlier treatment are also needed to reduce mortality in this setting [41].

This study shows that even though new AIDS events and death are uncommon in patients on cART with a suppressed viral load, these patients still benefit from a higher CD4 cell count. There is support in this study for starting cART when a patient's CD4 cell count is between 350 and 500 cells/µl and for continued vigilance when treating patients with sustained viral suppression but a low CD4 cell count.

## Supporting Information

Text S1 **Appendix: Patient selection and sensitivity analyses.**
(DOC)

## Abbreviations

cART: combination antiretroviral therapy
COHERE: Collaboration of Observational HIV Epidemiological Research in Europe
HR: hazard ratio
IQR: interquartile range
NRTI: nucleoside (or nucleotide) reverse-transcriptase inhibitor
