# Supplementary material for: CD4 Cell Count and the Risk of AIDS or Death in HIV-Infected Adults on Combination Antiretroviral Therapy with a Suppressed Viral Load: A Longitudinal Cohort Study from COHERE
Source: PLoS Med. 2012 Mar 20;9(3):e1001194. doi: 10.1371/journal.pmed.1001194 (PMC3308938; doi:10.1371/journal.pmed.1001194)
Supplement: Text S1 — Appendix: Patient selection and sensitivity analyses. (DOC) [file pmed.1001194.s001.doc]

# Supporting Information: Appendix

## Patient selection

Of the 176,585 patients in the 2010 merger of COHERE, antiretroviral treatment information was available for 149,302 patients. After excluding patients with obvious inconsistencies, 147,302 patients had a consistent treatment history and of these patients, 118,877 had at least one suppression episode where an episode was defined as starting with the first of two consecutive viral load measurements below 400 copies/ml; 105,835 patients had at least one such suppression episode while on cART. However this definition of a suppression episode would lead to an underestimate of mortality rates while suppressed (Table 2) because patients cannot die between their first and second viral load measurements. With a suppression episode defined as starting with the second of two consecutive viral load measurements below 400 copies/ml, 95,454 patients had at least one suppression episode while on cART. Optimal viral suppression is defined as a viral load below the level of detection or below 20–75 copies/ml depending on the assay used [5]. With a suppression episode defined as starting with the second of two consecutive viral load measurements below 50 copies/ml (or undetectable), 76,196 patients had at least one suppression episode while on cART. For 75,336 patients, a CD4 cell count was measured within 6 months prior to the start of an episode or within an episode; intervals from these episodes were used to calculate event rates (Table 2). Among these 75,336 patients, 66,147 patients had a viral load measured prior to starting cART; intervals from these episodes were used for the main analyses (Table 3).

To assess whether our results were influenced by this process of patient selection, we carried out additional sensitivity analyses.

## Planned sensitivity analyses

We carried out six planned sensitivity analyses to check that our model estimates were stable. Assays have become more sensitive over time, so we re-fitted our model with (1) a suppression episode re-defined as a viral load below 400 copies/ml – to simulate constant use over time of a less sensitive assay; and (2) with the analysis restricted to suppression episodes starting after 1 January 2001 – to largely omit episodes found using less sensitive assays. We varied the period of time after a last viral load measurement within which new AIDS events or death were accepted as outcomes if suppression was ongoing. We considered such events as outcomes if within 180 days of a last viral load where the patient was still suppressed at this last measurement, but in sensitivity analyses we re-fitted our model assuming (3) shorter and (4) longer periods (90 and 270 days respectively). We dropped covariates from our model to retain patient episodes lost from our analyses because of missing covariate values. We re-fitted our model (5) without viral load as a covariate because for many patients, we did not have a viral load measured prior to starting combination antiretroviral therapy (cART); and (6) without co-infection with hepatitis as a covariate, because then we could include additional patients in our analysis from the 2008 merger of the COHERE database.

The CD4 cell count gradient seen in the main analysis was also apparent in all six sensitivity analyses (Table A1). The main analysis was based on 1838 first new AIDS events or deaths. In sensitivity analyses, the number of these events ranged from 1234 when only episodes after 1 January 2001 were included in the analysis to 2430 when hepatitis was dropped as a covariate and data added from the 2008 merger. Hazard ratios for the spline representing CD4 cell count varied little in the six sensitivity analyses: 0.32 to 0.38, 0.78 to 0.86, 0.73 to 0.75 and 0.95 to 0.97 for the four spline components respectively.

## Additional sensitivity analyses

In an unplanned sensitivity analysis, we added an additional covariate to the analysis of the primary outcome, either taking value zero for a first suppression episode and one otherwise, or taking value zero for a first suppression episode and the period elapsed between episodes (in years) otherwise. We also estimated the loss in CD4 cell count between the end of one suppression episode and the beginning of the next in those patients with more than one episode of viral suppression while on cART.

The estimates for these additional covariates (HR 1.07, 0.95-1.20 and HR 1.02, 0.98-1.07, per year respectively) suggest that, having adjusted for other covariates (including a time updated CD4 cell count), patients with episodic suppression were no more likely to progress to a new AIDS event or death than patients with continuous suppression. Among the 75,336 patients with at least one suppression episode, 21,662 patients had more than one episode and contributed 28,929 periods between one suppression episode and the next. The estimated loss in CD4 cell count between the end of one suppression episode and the beginning of the next was 23 cells/µL per year (Figure A1). However, for 58% of these periods between episodes, patients were still on cART at the end of the previous episode. The estimated loss in CD4 cell count was 16 cells/µL per year in those still on cART at the end of the previous episode, and 37 cells/µL per year in those no longer on cART at the end of the previous episode. Note that patients no longer on cART might still be receiving one or two antiretroviral drugs, but not three. These estimates are likely to underestimate the decline in CD4 cell count after an suppression episode ends (and hence underestimate the consequences of a period of viremia) because patients with a greater decline are less likely to have another suppression episode.


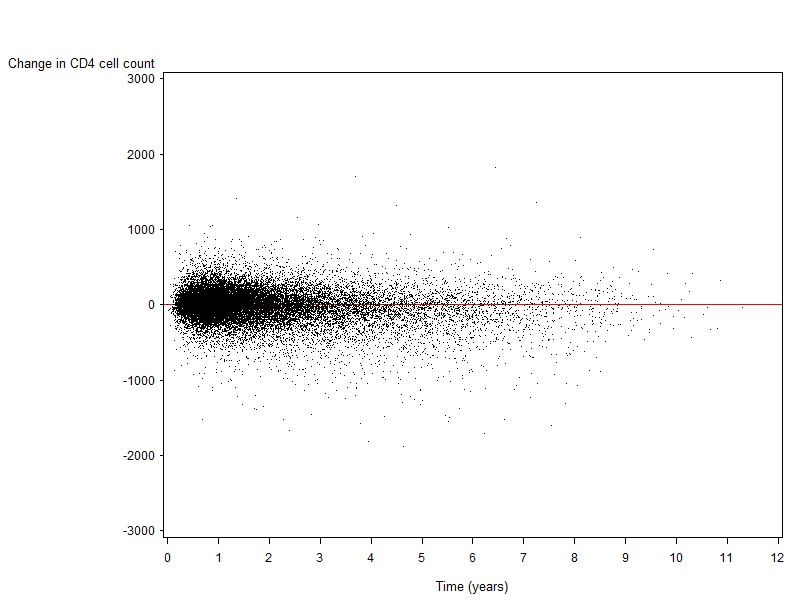


Figure A1: Change in CD4 cell count (in cells/µL) and the time (in years) between the end of one suppression episode and the beginning of the next. The change in CD4 cell count is negative if the CD4 cell count at the end of one episode is greater than the CD4 cell count at the beginning of the next episode.

Table A1: Main and six sensitivity analyses for the primary outcome (progression to a first new AIDS event or death) using multivariate Cox proportional hazard models. All analyses adjusted (as in Table 3) for age, gender, infection by drug use, hepatitis B or C, number of prior cART regimens, maximum viral load prior to suppression, and cART regimen.

|  | | Number of events | Hazard ratio estimates and their 95 % confidence intervals for  CD4 cell count (per 100 cells/µL) represented by a linear spline ^1^ | | | | | | | | | |
| --- | --- | --- | --- | --- | --- | --- | --- | --- | --- | --- | --- | --- |
| Analysis | |  | 0 - <200 | | 200 - <350 | | | 350 - <500 | | | ≥ 500 | |
| 0 | Main analysis (Table 3) | 1838 | 0.35 | 0.30-0.40 | | 0.81 | 0.71-0.92 | | 0.74 | 0.66-0.83 | 0.96 | 0.92-0.99 |
| 1 | Suppression defined by viral load <400 (instead of <50) copies/ml | 2422 | 0.38 | 0.34-0.41 | | 0.78 | 0.69-0.87 | | 0.73 | 0.66-0.81 | 0.97 | 0.93-1.00 |
| 2 | Suppression episodes starting after 1 January 2000 | 1234 | 0.32 | 0.26-0.38 | | 0.86 | 0.73-1.01 | | 0.73 | 0.64-0.84 | 0.97 | 0.92-1.01 |
| 3 | Events accepted within 90 days of last viral load if suppression ongoing | 1529 | 0.34 | 0.29-0.41 | | 0.82 | 0.71-0.95 | | 0.75 | 0.66-0.85 | 0.95 | 0.91-0.99 |
| 4 | Events accepted within 270 days of last viral load if suppression ongoing | 1948 | 0.36 | 0.31-0.42 | | 0.79 | 0.70-0.90 | | 0.73 | 0.65-0.82 | 0.96 | 0.92-0.99 |
| 5 | Without viral load as a covariate | 2257 | 0.35 | 0.30-0.40 | | 0.79 | 0.70-0.89 | | 0.73 | 0.66-0.82 | 0.97 | 0.94-1.00 |
| 6 | Without hepatitis as a covariate (and including data from 2008 merger) | 2430 | 0.35 | 0.31-0.40 | | 0.79 | 0.70-0.88 | | 0.74 | 0.67-0.82 | 0.96 | 0.93-0.99 |
| Abbreviations: cART, combination antiretroviral therapy.  ^1^ Time dependent covariate. A hazard ratio <1.0 for any of the four components of this spline implies that a higher CD4 cell count (per 100 cells/µL) is associated with a lower risk of progression and is therefore a measure of the benefit that a patient can expect if their CD4 cell count increases above any current level within the range covered by that spline component. | | | | | | | | | | | | |
